# Supplementary material for: Scale and information-processing thresholds in Holocene social evolution
Source: Nat Commun. 2020 May 14;11:2394. doi: 10.1038/s41467-020-16035-9 (PMC7224170; doi:10.1038/s41467-020-16035-9)
Supplement: Supplementary file 1 — Supplementary Information [file 41467_2020_16035_MOESM1_ESM.pdf]

# Supplementary Information for "Scale and Information-Processing Thresholds in Holocene Social Evolution" by Jaeweon Shin et al.

## Contents

**1 Supplementary Note 1: Overview of Seshat** **1**
1.1 Choice of NGAs . . . . . . . . . . 2
1.2 Choice of Variables and Byproducts of Imputation . . . . . . . . . . 2
**2 Supplementary Note 2: Previous Seshat Research** **3**
2.1 Evolution of Information Systems . . . . . . . . . . 3
2.2 Evolution of Specialized Governance Structures . . . . . . . . . . 4
**3 Supplementary Note 3: Bimodality** **4**
3.1 Gaussian Mixture Model . . . . . . . . . . 5
**4 Supplementary Note 4: Possible non-social science explanations of the bimodality** **8**
4.1 Discrete Markov transition model . . . . . . . . . . 8
Model Details
4.2 Saturation of variables . . . . . . . . . . 11
4.3 Undersampling . . . . . . . . . . 11
**5 Supplementary Note 5: Plots of PC1 by Time** **12**
**Supplementary References** **20**

## 1 Supplementary Note 1: Overview of Seshat

The Seshat project was initiated in 2009 to develop "a general resource for testing theories about socio-cultural evolution and cliodynamics" [1, p. 59]. Here we provide an overview of the Seshat database including its structure (1.1) and variables (1.2), identifying some issues raised by the imputation procedure in [2]. Seshat is an evolving database, and so this summary largely refers to the version of Seshat used in [2]. In section 2 we briefly summarize two more recent Seshat publications. The remainder of this SI analyzes the source(s) of the bimodality in polity scores on PC1 (section 3) and for reference plots the PC1 scores through time for each NGA (section 5).

The Seshat database divides the world spatially into distinct "Natural Geographic Areas" (NGAs) and then temporally subdivides these by "polities" each of which lasts for one or more centuries. An NGA is a purely geographic unit, defined as an area roughly 100 km by 100 km "delimited by naturally occurring geographical features (for example, river basins, coastal plains, valleys, and islands)" [2, p. 6]. Examples of NGAs are the Cambodian Basin, the Konya Plain, and the Big Island of Hawaii. Each NGA is controlled by a sequence of polities, with at most one polity controlling the NGA at any time. A polity is "an independent political unit that ranges in scale from groups organized as independent local communities to territorially expansive, multi-ethnic empires" [2, p. 2]. Seshat uses a representative quasi-polity when more than one political entity existed in an NGA at one time, or when control of the polity switched rapidly among political entities – see[1] for further details. Following Seshat usage[2,3], we do not distinguish quasi-polities from polities in our analysis.

Generally, polities are defined more granularly than in colloquial speech, and a new polity is assigned when there is a significant sociopolitical realignment. For example, Seshat recognizes eight polities in the Kingdom of France from Charlemagne to Louis XIV. Seshat encodes measurements of societal complexity (where available) at 100-year intervals for each NGA and its associated controlling polity. The relationship between NGAs, centuries, and polities can be clarified by the example of Iceland, an NGA. There are four observations for Iceland, one every century from AD 1000-1300 inclusive. The first three observations (AD 1000-1200) are coded as a single polity (the Commonwealth), and the last (AD 1300) as a separate polity (part of the Danish Kingdom).

### 1.1 Choice of NGAs

A subset of NGAs in Seshat was used in the analysis of Turchin and colleagues[2]. This set was chosen to maximize geographic extent and diversity in social organization. In particular, 30 NGAs were sampled from 10 world regions (e.g., South America; Africa). From each world region three NGAs were selected to provide one example each of a place where complexity appeared early, intermediate, and late in date. The resulting dataset contains 414 distinct observations across NGAs at 100-year intervals. Seshat is an evolving dataset with the versions resulting in publications available for download at http://seshatdatabank.info/datasets/.

### 1.2 Choice of Variables and Byproducts of Imputation

The Seshat database now contains over 1500 variables. However, only a subset was used for recent articles analyzing complexity (and by us). Turchin and colleagues[2] chose 51 variables related to social complexity that they believe can be reliably coded even for (well-known) archaeological contexts. These variables are imputed into a set of nine distinct summary variables, called "complexity characteristics" (CCs). The nine CCs measure: (1) polity population, (2) extent of polity territory, (3) population size of largest urban center, (4) hierarchical complexity (administrative, religious, military, settlement), (5) government, (6) infrastructure, (7) information systems, (8) specialized literature, and (9) monetary system (see also Table 1, main text).

A problem with this imputation is that there are frequently one or more of the original variables for which values are missing. This can be due to incomplete research, expert disagreement, or other uncertainties about the correct value. Missing values are especially common for earlier polities for which less information is available. Rather than assign a value and create one canonical imputation, instead a distribution of possible values is assigned. These distributions are then sampled 20 times, to produce 20 replicates of the dataset [2, p. 7]. The imputation is performed on each replicate, producing 20 different sets of CC values. These replicates are used to produce confidence intervals on the proportion of variance explained by each PC, the component loadings, and the values of the PCs for each polity. The authors graciously provided the 20 imputed datasets, forming the basis of our analysis here. The complete dataset contains 8280 rows (414 observations $*$ 20 imputations), and 13 columns. The columns are: the name of the NGA, an identifier for the polity, the century of the observation, the value for each of the nine CCs (the result of imputation), and the imputation replicate identifier.

It is visually apparent that there are artifacts in the data shown in Supplementary Figure 2. The data exhibit noticeable "streaks" at approximately a thirty-degree angle from the vertical. We examined the cause of these streaks to ensure they are not a significant contributor to our findings. We first examined whether the streaks are caused by strong correlations among (a set of) CCs. We examined this by reproducing Supplementary Figure 2 but zeroing out the contribution from all combinations of 1, 2, and 3 CCs.

The character of the streaks (primarily the orientation) changes when certain CC combinations are

removed, but all subsets still contain visible streaks. This implies the streaks are not caused by strong CC correlations. It also implies they are not caused by the fact that some CCs are more "discrete" than others, for instance measured in integer increments from 1 to 5 rather than a more continuous measure. Instead, these streak artifacts are caused by the procedure used by[2] to generate the 20 dataset replicates. Each streak is, in fact, a single polity with its 20 replicates. The apparent directionality of the streaks arises from the uneven uncertainty assigned to each CC. Population measures, for instance, are specified in a range that is sampled, while Money Level CC was not assigned an uncertainty. In between are multiple uncertainties that are averaged into a single CC, and so their combined variance is reduced compared with population. These streaks give a visual representation of the effective uncertainty assigned to each polity. We find that the measured uncertainty in the Seshat data does not impact the results of the analysis in this work, nor the analysis of Turchin and colleagues[2]. The variation in each polity is small compared with the variation between polities. This level of measured uncertainty does not affect the existence of two strong clusters, and this noise, if anything, reduces the strength of the primary principal component rather than enhancing it. We incorporate the uncertainty from the different imputations in reporting the rest of our results.

## 2 Supplementary Note 2: Previous Seshat Research

We do not attempt a comprehensive review of the rapidly growing set of articles from this project. Instead, we summarize three recent, relevant articles. The foundational article in this line of inquiry[2] describes and analyzes the dataset we consider here. The major finding is the primary principal component (PC) captures 77% of the variation in the "complexity components" (CCs), with approximately equal loading by all nine CCs onto that first PC. Small-scale polities (typically earlier in time) have generally low scores when projected onto this primary PC, while larger-scale polities (typically later in time) generally have a high projection (score). The location of any single polity along this one, nine-dimensional PC might plausibly serve as a useful scalar measure of how complex, or possibly "developed", that polity was, in the sense of sharing features with large-scale rather than small-scale societies.

### 2.1 Evolution of Information Systems

The time-stamping of each observation is used profitably in two other recent papers from the Seshat group. The first[4] uses multiple linear regression to explain variation through time in the information complexity CC (*Info*). Potential independent variables lead the dependent by one century so that causal inferences respect time's arrow. There is also an autoregressive term for the dependent, to examine the extent to which earlier values for *Info* predict the current value. Finally, there are terms examining correlations due to shared culture history (proxied as language family) and spatial diffusion. The best linear model (that with the smallest AIC) identified first- and second-order lags of *Info* to be the most important predictors of *Info*, followed by phylogeny, *Money*, and *PolPop* (polity population), all with positive slopes. *Money* is a scale reflecting the "most sophisticated" monetary instrument present in the coded polity (0: none, 1: Articles, 2: Tokens, 3: Precious metals, 4: Foreign coins, 5: Indigenous coins, 6: Paper currency) [4, p. 39]. Broadly similar results were achieved when quadratic (squared) terms for the regressors were introduced although the Lag1 squared term for the autoregressive version of the independent variable bore a negative sign, possibly suggesting a "regulatory" effect for this term: "when *Info* gets too high, this term "steps on the brakes" to impede its further increase" [4, p. 51].

## 2.2 Evolution of Specialized Governance Structures

Another recent paper[5] employs the same approach to analyze the evolution of the internal specialization of governance structures (*Gov*) which sums 11 binary variables reflecting the presence/absence of various specific offices, government buildings, examination systems, etc. [5, p. 3]. Here, the lag1 version of *Gov* is by far the most important variable in the nonlinear regression, followed by the phylogeny proxy, a quadratic version of *Gov*, and (log-transformed) polity population (all signs positive except for *Gov*). The only other independent variable with a significant $t$ value is *Time*, the absolute date of each time step. The authors interpret the inclusion of this variable in all the best-fitting models to indicate that important unobserved variables remain to be discovered and added to the model.

As valuable as these two analyses are, particularly since they take time explicitly into account, they deal with just one (dependent) variable at a time. Our re-analysis of the data in the main paper[2] where all the variables are considered simultaneously, is designed to see what gains can be made when that analysis is performed in a dynamic mode.

# 3 Supplementary Note 3: Bimodality

Our reanalysis of the Seshat dataset reported by Turchin[2] was originally motivated by the apparent bimodality in the frequency of polities projected onto their PC1 as illustrated in Supplementary Figure 1. This was first of all a concern: the primary formal justification for an analysis based on PCA (as in[2]) is the assumption that the data are generated by IID sampling a *single* Gaussian. The presence in these data of two highly distinct clusters is therefore problematic. Indeed, as shown in Supplementary Figure 1, the clusters in PC1 appear very similar, being accurately described by a mixture of *two* Gaussians of very similar widths, distinguished only by their mean values on PC1. Other results emerging from the PCA, such as the finding that PC1 captures "77% of the variability" become unreliable given this data structure. That is one justification for our decision to pay more attention to PC2, since, likewise, it may be more important than the statistics generated in[2] suggest. Accordingly, as a conservative first step, in this paper we consider the Seshat dataset projected onto the top two PCs.

The second reason the bimodality is of interest however is that — if not an artifact of sampling or analysis — it may suggest a fundamental division of societies, perhaps into "simple" and "complex" groups, that additionally might be regarded as fundamental basins of attraction for the dynamics of social groups as they move through the Holocene. (For example, on the basis of a somewhat similar analysis Peter Peregrine[6] has recently argued for the existence of four modes in sociopolitical organization.) One of these clusters (with a modal score around -4 on PC1) corresponds to polities of modest demographic scale, often but not invariably early in time. The other cluster (with a modal score around 2 on PC1) corresponds to polities of larger demographic scale, generally appearing later in time. As a shorthand we will refer to these as the "early" and "late" clusters, though this is not invariably true in calendar years. The only polities that reach the scale required for stable membership in the late cluster, at least in this sample, are from the Old World (Eurasia, Africa, and Oceania).

Turchin and colleagues[2] also recognized these two clusters. On pp. 26-28 of the supplemental information they note "some initial support for the idea" of clusters in the distribution of societies along PC1 "with a relatively large distance between two main clusters". Figure SI11 in that paper suggests that two modes begin to develop along the PC1 dimension by 1000 BC and are quite prominent by AD 1. Similarly, Turchin et al. [5, p. 13] report that "there may be two clusters in the governance–population phase space, where trajectories tend to spend most time. The transition between the two clusters, on the other hand, happens quickly."

We now present several analyses that suggest that the bimodal distribution of points along PC1 is

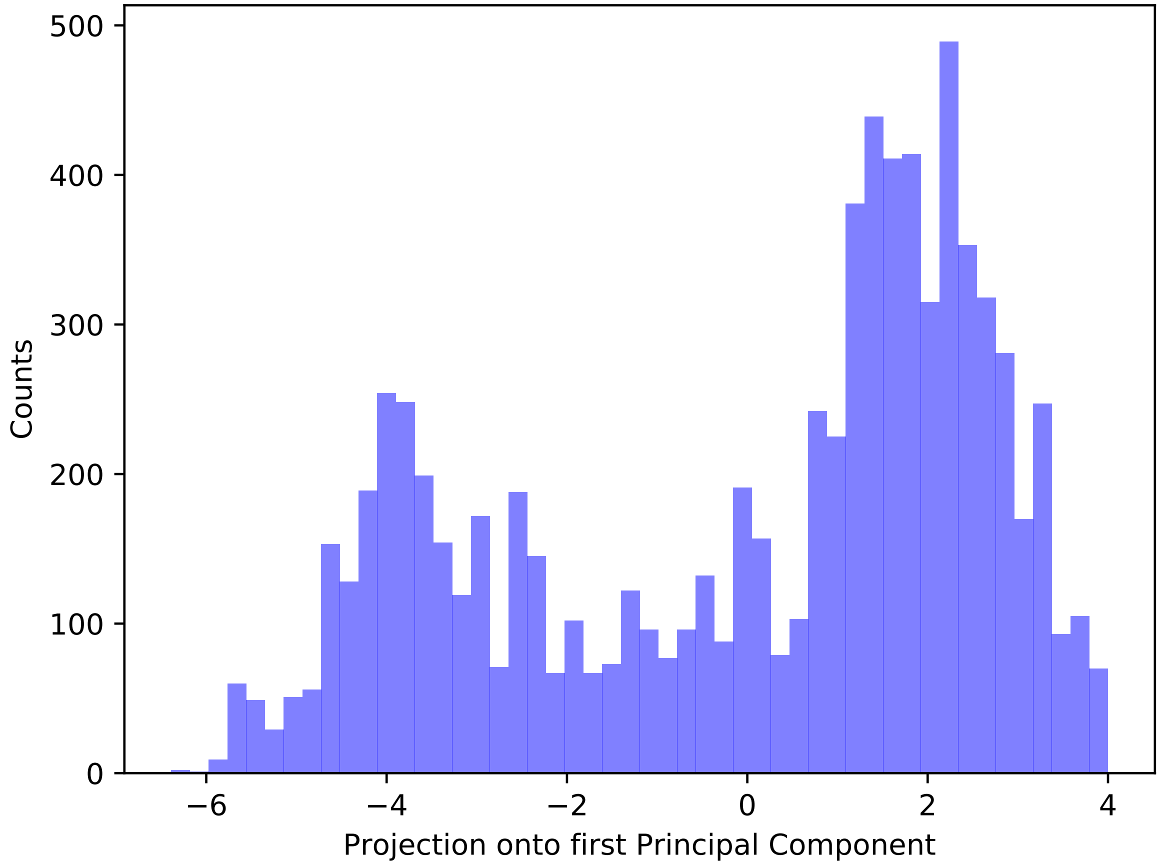


**Supplementary Figure 1.** Histogram of polities projected onto PC1. Each imputation comprises a separate point in the underlying data for the histogram.

likely not of substantive interest, though this structure still raises issues for the strict interpretation of the proportions of variance explained by the various PCs. In particular, the bimodality is *not* the result of the streak artifacts discussed in 1.2. We also show that the bimodality is not the result of saturation of the variables recorded in the Seshat dataset at their extremal values as societies develop. (Such saturation may however contribute to the accumulation of datapoints in the right-hand cluster). There were also some possibilities we could not reject. In particular, we could not conclude that there is not an attractor of the underlying dynamics (i.e., a “sink”) in the right-hand cluster.

### 3.1 Gaussian Mixture Model

To investigate the bimodality in more detail we began by fitting a two-component Gaussian mixture model to the full 9-dimensional (9D) imputed Seshat dataset. Supplementary Figure 2 presents a 2D scatterplot of PC1-PC2, with the coloring of the points determined by which 9D Gaussian cluster each point most likely belongs to. We define cluster membership for each point as its maximum likelihood cluster, i.e., the Gaussian with the higher membership probability. (Note that the true data are 9-dimensional, and the full data are used to define membership, given the seemingly non-monotonic clustering classification.)

Moreover, as shown in Supplementary Figure 3, most of the data points are included in the $< 99\%$

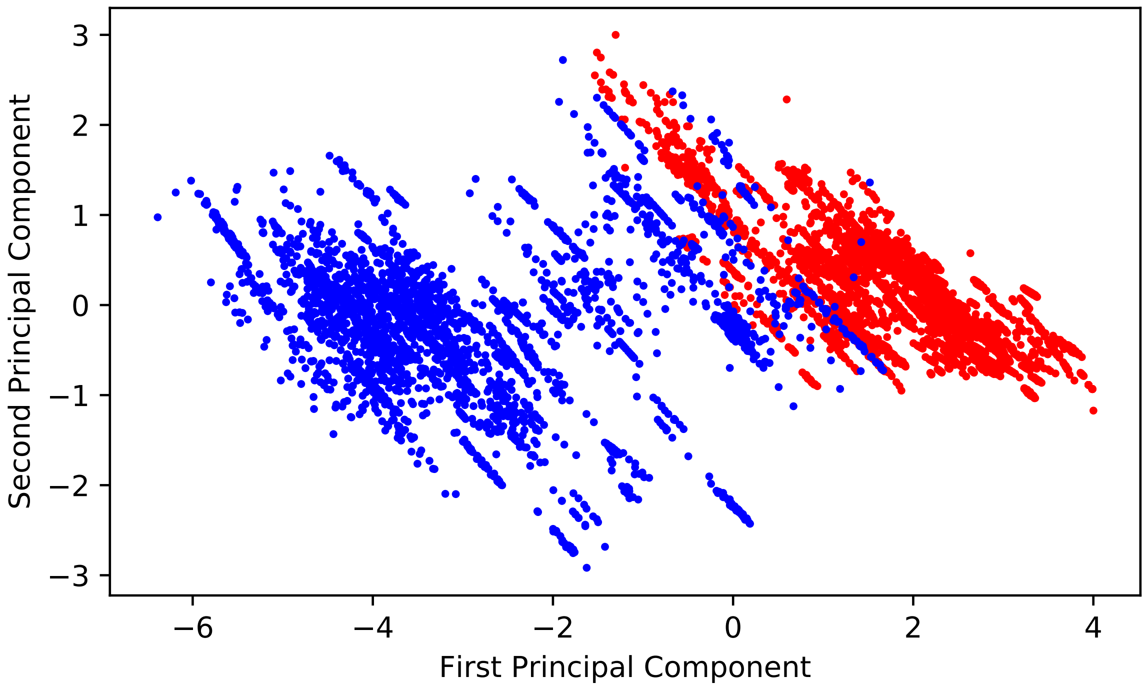


**Supplementary Figure 2.** Scatterplot of polities projected onto the first and second PC. The left (blue) and right (red) clusters are generated by fitting a two-component Gaussian mixture to the 9-dimensional PC data and determining, based on the mixture densities, whether a point belongs to the first or second cluster. The overlap between points in the cluster occurs because the 9-dimensional fit is displayed projected into the 2-dimensional PC1-PC2 space. In fact, a point belongs exclusively to one of the two clusters as suggested by Supplementary Figure 3. Note the pronounced "streaking" from top left to bottom right which appears to be due to the imputation procedure.

threshold, meaning that most points exclusively belong to one of the two clusters according to our Gaussian Mixture Model. This result suggests that the two clusters we observed in the data are clearly distinguishable.

To test whether the two Gaussian clusters were due simply to noise, we bootstrapped the following quantities: 1) the percentage of the sum of all eigenvalues that is given by the largest eigenvalue alone, in the first and the second Gaussians, 2) the angle between the principal axes of the two Gaussians, corresponding to the largest eigenvalue in each Gaussian, and 3) the angle between PC1 and the principal axis of the first Gaussian, and the angle between PC1 and the principal axis of the second Gaussian. Note that all these values were obtained by fitting two Gaussians in the 9-dimensional imputed Seshat data matrix.

In more detail, the these properties of the Gaussians were calculated as follows. 1) To compute

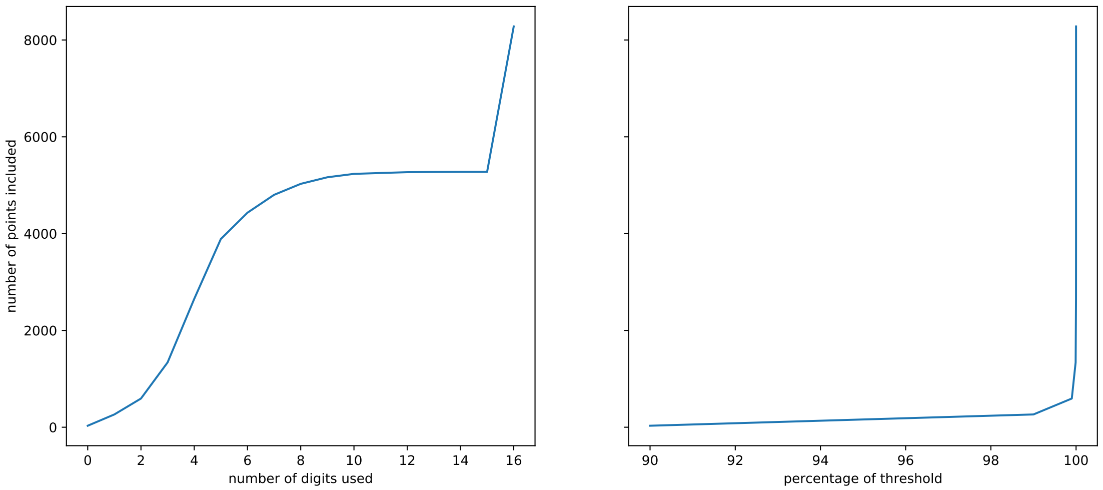


**Supplementary Figure 3.** Result for verifying the robustness of the two Gaussian clusters. After fitting the Gaussian mixture model with two components, using the predicted posterior probability for each component for each data point, we determine how many points have above the given threshold probability of belonging exclusively to one of the two components. Left: total number of points included in either of the clusters based on the number of digits used for the threshold probability (e.g., 6 digits for the threshold means that the threshold was 0.999999). Note that in this left-hand plot, when the number of digits used for the threshold reached 16, every data point was included in the clusters due to numerical overflow error. Right: number of points included in the two clusters when changing the threshold of the probability value (quantified as a percentage) for belonging exclusively to one or the other of the two clusters (e.g., "90 percent threshold" picks out data points that have less than 90% probability of belonging exclusively to either of the two clusters).

the percentage of the largest eigenvalue in the two Gaussians, we first calculated 9 eigenvalues for the covariance matrix of each of the two fitted Gaussians. For each Gaussian, we then selected the eigenvalue with highest value and divided it by the sum of all 9 eigenvalues to obtain the percentage of the largest eigenvalue. 2) To compute the angle between the two main eigenvectors, we selected the eigenvector that corresponded to the largest eigenvalue in each of the two Gaussians. Then, we calculated the angle between the two vectors using the cosine formula. 3) Computing the angle between Turchin's main principal axis and the main eigenvectors of the first and the second Gaussian follows exactly the same procedure as above, by taking the main principal axis vector and computing the angle between that vector with the main eigenvectors of the two Gaussians.

The procedure for bootstrapping was as follows. First, for each of the methods listed, we randomly resampled the original 9-dimensional imputed data matrix and obtained 5000 matrices that are of the same dimension as the original matrix. Then, for each of these 5000 matrices, we computed the statistic of our interest (e.g., angle between the two main eigenvectors) and obtained an array of resulting values for the statistic. We plotted the values calculated from resampled matrices, as well as plotting a vertical line that indicates the value calculated from the original matrix. Furthermore, we indicated the upper and lower bounds of the bootstrap normal 95%-confidence interval with dashed lines, which were computed as follows: suppose that for an estimate $T$ of interest, bootstrap estimates are given by $T_1, \ldots, T_n$ with original sample estimate $T_0$. Then, the normal 95%-confidence interval is given by $T_0$ - bias $\pm$ $std(T_1, \ldots, T_n)$,

where bias is defined as $mean(T_1,...,T_m) - T_0$ and $std(T_1,...,T_n)$ refers to the standard deviation of the bootstrapped estimates. The result (Supplementary Figure 4) suggests that the outcome is robust, implying that the two Gaussians were not caused by noise.

So we wish to ask whether this the bimodality is of substantive interest, or some kind of an artifact of the statistical analysis or the data-generation methods. (Note that some of those data-gathering methods are implicit, arising in how archaeologists choose where to excavate and what variables to pay attention to. Others are explicit, for example the decision by the creators of Seshat to exclude many polities from the database to try to get uniform coverage over NGAs, or of the use of imputations to "fill in" holes in that database.) We examined several candidate hypotheses for the bimodality and were able to statistically reject several of them — but not all. Some of these null hypotheses are discussed in the next section.

# 4 Supplementary Note 4: Possible non-social science explanations of the bimodality

Imagine for a moment that the time-stamping of all the polities were randomly re-assigned while keeping all the other attributes for each polity intact. A PCA would still "explain" 77% of the (static) patterning in the data, but each polity's movement along the continuum defined by PC1, over time, would be random. In that case, would we think that PC1 provides a useful summary of sociopolitical development? Probably not, since we understand (sociopolitical) evolution as an historical process. What really counts for understanding such a process is not the "static pattern" formed by superimposing a sequence of snapshots of the process. Rather we ought to be interested in the dynamics of that entire sequence. This observation alone warrants examination of the dynamics of the data, ideally in more dimensions than just PC1. Indeed, as described below, many aspects of static patterns can be misleading, leading to conclusions that in fact are contravened if one directly analyzes the dynamics. This issue is not assessed by[2] (except for noting after having performed the PCA that early polities tend to have lower values of PC1 than later polities).

## 4.1 Discrete Markov transition model

To more directly address whether the dataset reflects variation in the underlying dynamics as one moves across PC1 or just a static pattern, we constructed a novel null-hypothesis technique. This technique is based on the observation that even if the underlying dynamics through PC space is a time-homogeneous Markov chain, with no basins of attraction, and no dependence on time, the *dwell times* in the various parts of PC space caused by evolving under that Markov chain could vary significantly. That heterogeneity in dwell times might in turn cause the observed bimodality, ironically due to the fact that datapoints are sampled uniformly in time. In essence, the uniformity of sampling in time would introduce an artifact of *non*-uniformity of the distribution over space.

To investigate this hypothesis, we binned PC1 and then used the (time-stamped) Seshat data to estimate the conditional distribution of moving from one bin to another over a given (fixed) time interval, $\Delta t$. We then iteratively applied that conditional distribution to every datapoint in Seshat, *exactly* $t/\Delta t$ *times* (where $t$ is the number of years ago of the timestamp of that datapoint). We then compared the resultant distribution of points to the full Seshat dataset. This amounts to assessing the null hypothesis that the distribution of points in PC1 arises from a combination of the following phenomena:

a) Random variation across the NGAs in the PC1 value of the earliest polity within that NGA;

b) Random variation across the NGAs in the chronological time of that first PC1 value;

c) A general drift of polities from low to high PC1 values driven by a time-homogeneous Markov chain.

Our tentative conclusion is that the data are consistent with our null hypothesis, i.e., that these three phenomena, by themselves, can explain the bimodality in PC1. Moreover, the conditional distribution of next bin given current bin under the estimated Markov chain of item (c) is irreducible (by inspection — see below). So there is a unique fixed point of this null hypothesis dynamics. This indicates that there is no evidence for there being more than one "basin of attraction" of the dynamics.

In addition we performed a very preliminary analysis of the relationship between the precise details of a stochastic process traversing a space and the resultant static pattern given by sampling that process at regular time points (as was done in generating Seshat, where the sampling time points were one century apart). Obviously, if the expected speed of movement across PC1 is slow for a broad interval of PC1, then is fast in a following interval, and then slow again in a following, third broad interval, there would be an increased number of datapoints in that first broad interval as well as the third one, with few points in between (given that the datapoints are formed by sampling at times all one century apart). This would result in the kind of bimodality in PC1 we found in the Seshat dataset. Conversely, if mean speed across PC1 were uniform across PC1, *and all other aspects of the dynamics were also uniform*, there would be no such induced bimodality in the samples of PC1 values.

However, we realized that even if the mean speed is uniform across PC1, variation in the *breadth* of possible speeds from one point in PC1 to another can result in bimodality of the sort that characterizes the Seshat dataset. To see why, first note that the amount of time a given polity will spend in a given interval of PC1 as it moves across PC1 will depend on the *inverse* of the speed across that region. Moreover, if we have two distributions over speed values that are both Gaussian with the same mean, but one of those distributions has a bigger variance than the other, the associated expected *inverse* speed will be greater in the distribution with a larger variance of speeds. This means that there will be a greater density of points sampled from a region of the space with a high variance of speeds than from a region of the space with a low variance of speeds.[1]

Accordingly, as a variant of our null-hypothesis test of what explains the bimodality, we modified it to try to correct for this dependence of dwell times on the variance of speeds. We did this by modifying how we estimate the conditional distribution of the Markov chain, to force no such variation across PC1 in the variance of the velocity from one point in PC1 to another. More precisely, we first estimated the average over all the bins of the variance of the speeds leaving that bin, $\sigma^2$. Then for each column probability vector $p$ in the conditional distribution matrix of next bin given current bin, we solved for the distribution $q$ with minimal Kullback-Leibler divergence to $p$ which has its variance of speeds equal to the global average of such variances, $\sigma^2$. We then replaced $p$ by $q$. Doing this for all columns in the original conditional distribution matrix gives a new conditional distribution matrix, which we could then apply to the starting polity position / times of the datapoints in Seshat, just as before. This variant of the null hypothesis appears to give a slightly worse fit to the actual full Seshat data than the original null hypothesis, in which the variances of speeds are allowed to vary across PC1. This leads us to conclude that there is weak but inconclusive evidence that variation in the variance of the speeds of movement across PC1 contributes to the bimodality.

The details of these analyses are presented in the next subsection. Before presenting them though, it is important to emphasize that while the *static* bimodal pattern can be (mostly) explained as a combination of phenomena (a)-(c), the ultimate question for us is what patterns there are in the *dynamics* across the space. To a degree, this can be assessed by examining the Markov transition matrices made under our null

[1]As a simple illustration, if the speed of movement across a bin of width 1 is 1, with probability 1, then it will take 1 unit of time to traverse that bin. If instead the speed when traversing that bin is either 1.5 or .5 with equal probability, the mean speed is again 1, but it will take on average $(2+2/3)/2 = 4/3$ units of time to traverse the bin. So there would be a greater expected dwell time in that bin.

models. However, an important direction for future work is to use more sophisticated statistical techniques to tease out that dynamics from the limited data.

#### ***4.1.1 Model Details***

In light of the extremely small amount of data, we considered only the dynamics over PC1, and discretized those PC1 values into 10 bins. We then formalized the phenomena (a)-(c) in 4.1 with the following model:

1. NGAs are "born" (i.e., first possess polities of sufficient levels of social development to warrant inclusion in the Seshat database) by IID sampling a distribution $p_0$ over those 10 bins;

2. The chronological time of the birth of each NGA is given by IID sampling a distribution $\Delta(t_0)$;

3. All NGAs evolve across the bins over time-jumps of 500 years by sampling the same (time-homogeneous) conditional distribution $\pi$;

4. All data points, over all times, are subject to observational noise (including random removal from the dataset entirely) to generate the final dataset.

For simplicity, we estimated $\pi$ by applying a frequency-count estimator to the 500-year bin-to-bin transitions in Seshat (shown in Supplementary Figure 5). However, to be maximally conservative, we avoided estimating $\Delta(.)$ and $p_0(.)$, by pursuing the following procedure instead: First, for each NGA $n$, we found the earliest bin at which it is present in Seshat, $i(n)$. Let $T(n)$ be the number of 500-year blocks into the past of that earliest data point $i(n)$. We applied $\pi$ to $i(n)$ a total of $T(n)$ times, to generate a set of $T(n)$ bin values; doing this many times gave us a scatterplot of the PC1 values that we would expect to see for that NGA, if indeed its dynamics was given by $\pi$. Running this procedure for all of the NGAs gives an overall scatterplot which, if our model is correct, should look similar to the actual Seshat scatterplot. Supplementary Figure 6 (A) shows the results from this sampling procedure, which clearly exhibits bimodality similar to the original data.

We used this simple model to investigate the dynamic cause of the bimodality. Recall that the Seshat datapoints were recorded for regularly sampled times one century apart. This means that the two clusters would arise if movement through PC1-PC2 space was smaller where the first and second clusters lie, and faster in between them. In other words, it would arise if there were heterogeneity in the mean velocities across PC1-PC2 space. In terms of our discussion just above, this would correspond to variation, across the rows of the transition matrix $\pi$, of the difference between the average column index specified by the distribution of that row and the row index itself, i.e., variation with bin $n$ of the difference $\left[\sum_m \pi(m \mid n)m\right] - n$.

Perhaps more surprisingly, *even if the mean velocities do not vary*, if the *variance* in the mean velocities varies in the appropriate way, then clusters would arise in regions where that variance is relatively large. To see this, note that the amount of time an NGA stays in a given PC1 interval is proportional to its *inverse* speed across PC1 in that interval. Therefore a Gaussian distribution in speeds will result in a non-Gaussian distribution in the amount of time an NGA stays in a given PC1 interval, i.e., a non-Gaussian distribution in the number of Seshat data points in that interval.

To test these possibilities, we first generated a new transition matrix that resembles the original matrix $\pi$, except the mean and/or variance of speeds across the bins was forced to be the same, no matter what the originating bin. For each bin $n$, we generated the associated conditional distribution (i.e., row) of this alternative transition matrix, $\pi'(.|n)$, by minimizing the KL-divergence between $\pi'(.|n)$ and $\pi(.|n)$, subject to the constraints that the mean and/or variance of that new conditional distribution equals the associated value for the entire population, running over all $n$. Supplementary Figure 6 B, C, and D simulate

the histogram with constrained mean velocity, constrained variance in velocity, and both, respectively. The bimodality observed in the data (Supplementary Figure 6 A) is still present in all these graphs. This suggests that variation in the mean and/or variance of speed in different PC1 regions cannot be the sole explanation for the bimodality, though we do not exclude the possibility that such variations contribute.

In addition to these possible explanations of the bimodality grounded in dynamics through PC1-PC2 space, it is also possible that the bimodality is simply caused by the sampling process that generated the initial distribution. That is, in Seshat the initial observation of NGAs is biased towards low PC1 values, which by itself would create the early cluster, even without any superimposed dynamic process. To see this, we simulated the histogram by setting $p_0$ to be a uniform distribution i.e. equal probability across bins, while keeping the transition matrix $\pi$ the same as estimated. In Supplementary Figure 6 E, we see that the size of the left cluster is reduced significantly from what is actually found in the Seshat data. This implies that non-uniformity of the initial values of PC1 for each NGA explains the left cluster, at least in part.

### 4.2 Saturation of variables

There are several ways in which the observed bimodality could result from a statistical artifact in the sampling or definition of the data or variables. One scenario is that the two peaks are generated from saturation of the variables (the nine CCs). If the saturation of different variables occurs at different rates, then peaks would be observed around the time when each variable reaches its upper limit and the growth of PC is temporarily slowed.

To test for this, we tried some simple simulations where the PC is a sum of two variables, both of which follow logistic growth $(L_i/(1.+\exp(-k_i*(x-x_i^0))), i=1,2)$ but with different parameters. Supplementary Figure 7 (a)-(c) shows how the two variables with different saturation times sum up. Assuming polities that follow this growth path are sampled uniformly, the resulting histogram is shown in Supplementary Figure 7 (d). While the two peaks can be generated from this logic, the right peak is at the right-most time period, in contrast to the data graphed in Supplementary Figure 1. It is also worth noting that it requires us to fine-tune parameters to create such peaks. Supplementary Figure 8 shows different combinations of saturation parameters. In general, the uniform sampling results in a plateau at the right rather than two spikes if both of the variables are saturated within the observed period.

### 4.3 Undersampling

Another possible explanation for the bimodality is selection bias in the data. For example, perhaps polities of intermediate complexity were undersampled for some reason, such as lack of archaeological excavation or reporting for these polities, which might yield a bimodal distribution. To check that the bimodality (especially the peaks) was not caused by undersampling issues, we ran the following tests. Although it may be far-fetched to claim that these tests were sufficient to confirm that undersampling was not a problem, bimodality survived after each of these tests (Supplementary Figure 9).

1. Interpolating missing time periods within each NGA: We linearly interpolate PC1 by NGA to fill out the missing periods.

2. Weighting within each NGA depending on the sparsity of data within its neighborhood: For instance, say we observe an NGA at periods $t_1$, $t_2$, and $t_3$. We weigh the observation at $t_2$ by $\frac{(t_2-t_1)+(t_3-t_2)}{2}$ so that the observation in a sparse period counts more.

3. Use data points only from the NGAs that span both Gaussians: We take the NGAs that are classified as the first Gaussian in some periods, and the second Gaussian in other periods.

Supplementary Figure 9 plots the PC1 value by the normalized density for each of the scnearios above, linearly interpolating the main PC values for each NGA for missing periods; the two peaks remain for all these cases. We found the two clusters of points, each corresponding to peaks in the bimodal curve after doing a scatter plot under each method, with time and the main PC value as the two axes (not shown). Also, we found that there were fast transitions from one (lower) cluster to the other (higher cluster).

## 5 Supplementary Note 5: Plots of PC1 by Time

In the main text we provide several plots showing PC1 scores by PC2 scores. Supplementary Figure 10 provides plots of PC1 scores through time for each NGA. Note that if each of these 30 sequences exhibited a perfect positive correlation of its score on PC1 with its date, then we would be incorrect to maintain that the pattern-based correlational analysis undertaken by[2] cannot stand in for the dynamics. However, this is clearly not the case. Moreover, even if this were true, we see that these individual sequences exhibit very different slopes and intercepts — a fact of substantive interest though not pursued in this article. Of course we should be wary of putting too much emphasis on such a correlational analysis since some of the values in these figures are interpolated.

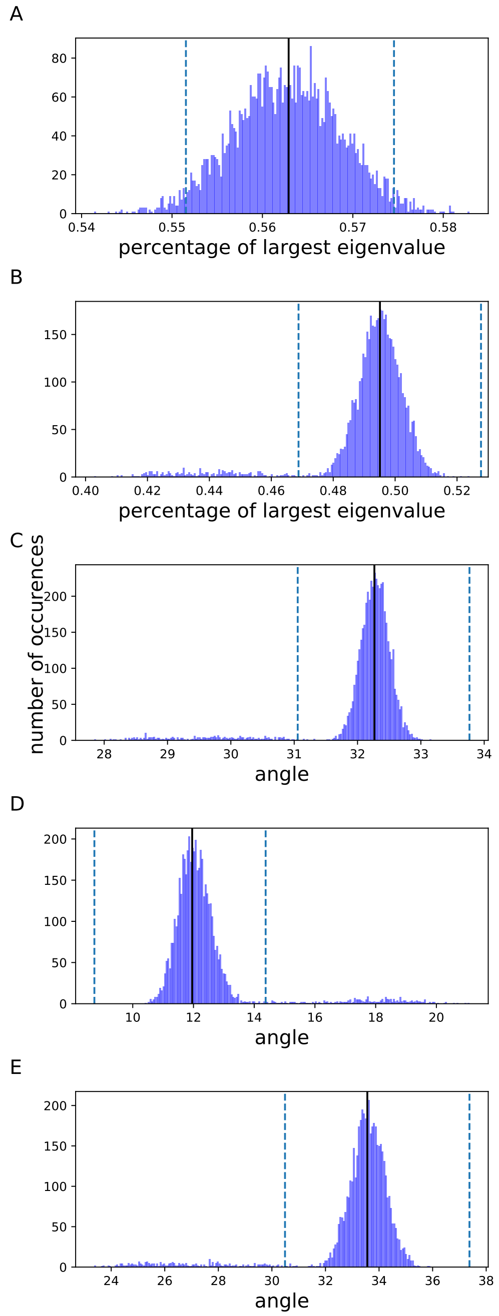


**Supplementary Figure 4.** Bootstrapping results for various statistics to verify the robustness of the two Gaussian clusters along with 95%-confidence interval. (A) The percentage of the sum of all eigenvalues in the first cluster given by the largest eigenvalue. (B) The percentage of the sum of all eigenvalues in the second cluster given by the largest eigenvalue. (C) The angle between PC1 and the principal axis of the first cluster. (D) The angle between PC1 and the principal axis of the second cluster. (E) The angle between the principal axes of the two clusters.

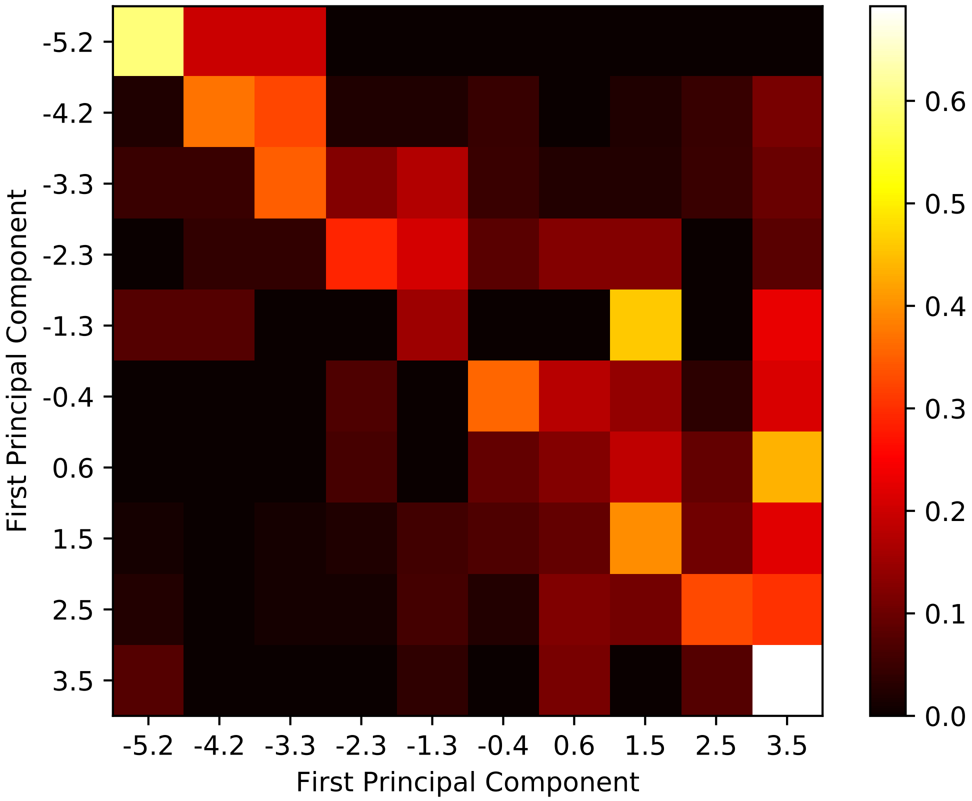


**Supplementary Figure 5.** The estimated transition probability $\pi$.

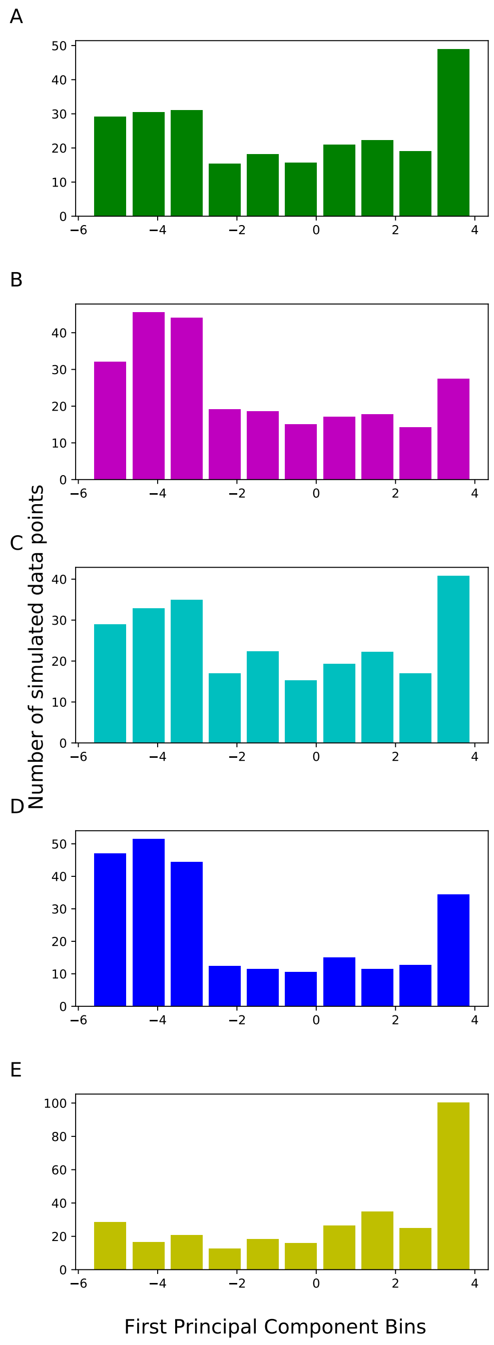


**Supplementary Figure 6.** Histograms of the simulated results from the Markov transition model. (A) The original data. (B) Constrained mean speed. (C) Constrained variance of speed. (D) Constrained mean and variance of speed. (E) Uniform distribution.

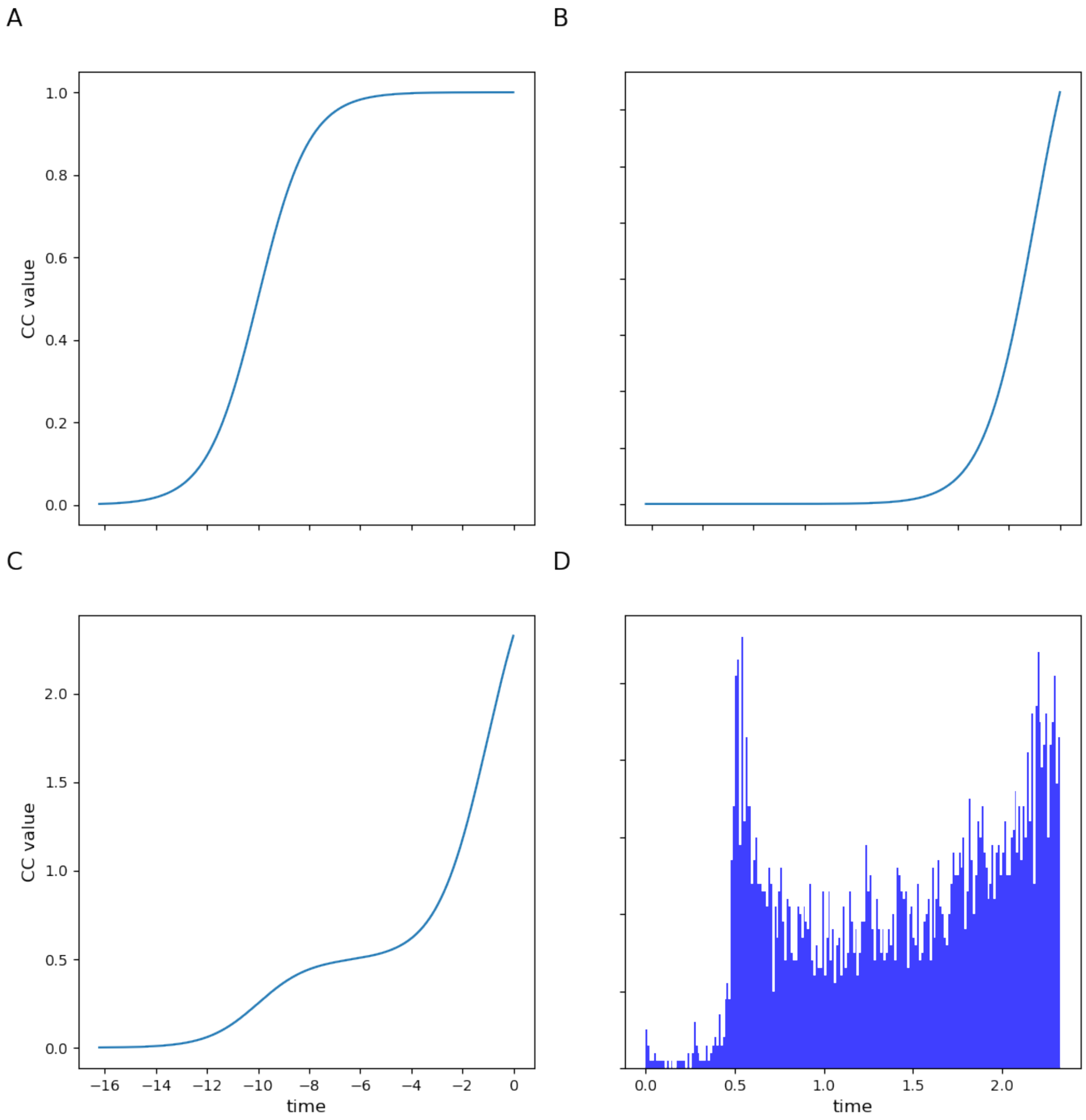


**Supplementary Figure 7.** The simulated logistic growth of two CCs. (A) First CC growth, (B) Second CC growth, (C) Growth of the average, (D) Histogram of the average under uniform distribution.

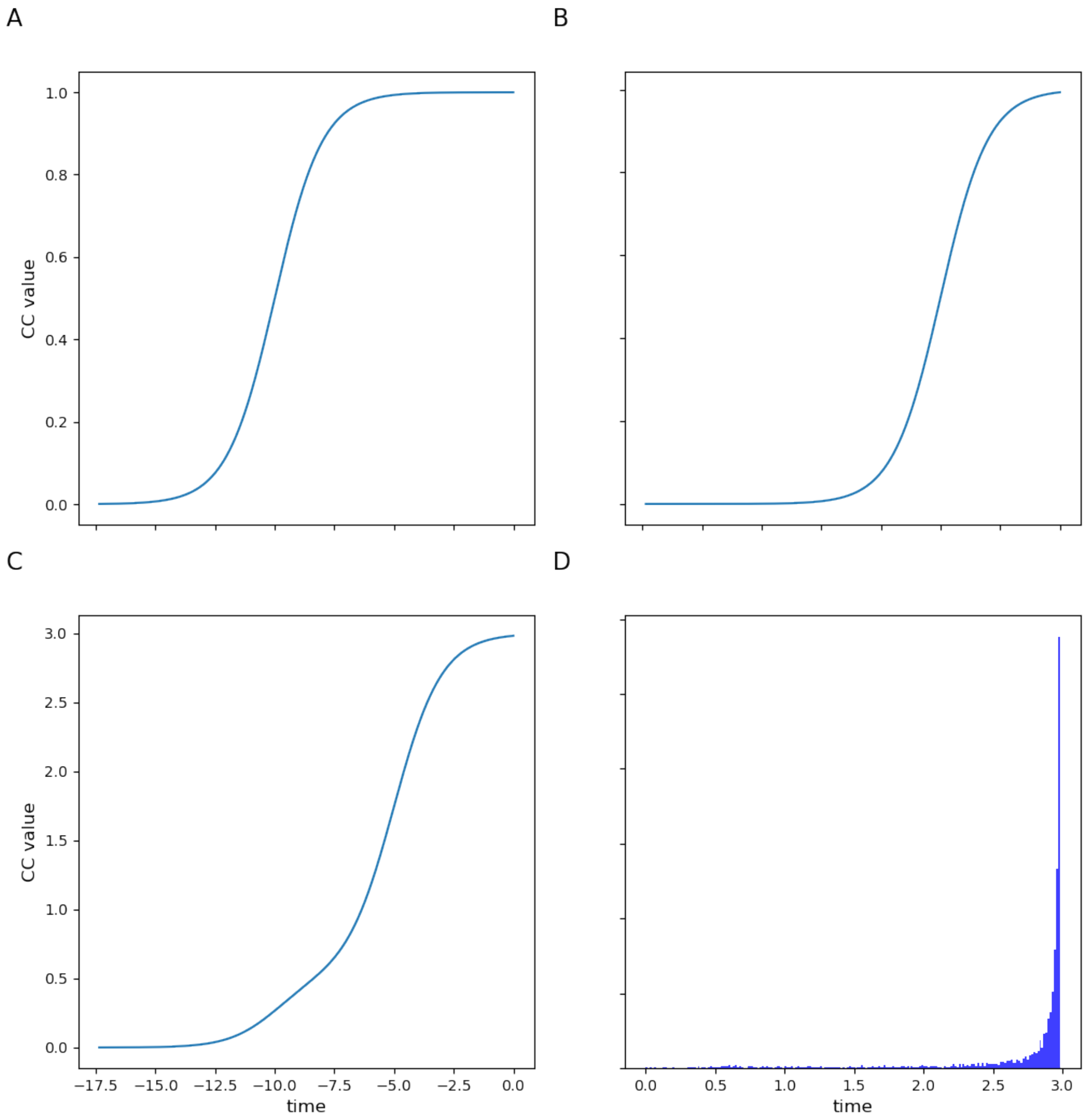


**Supplementary Figure 8.** The simulation of logistic growth CCs with different parameters. (A) First CC growth, (B) Second CC growth, (C) Growth of the average, (D) Histogram of the average of two CC.

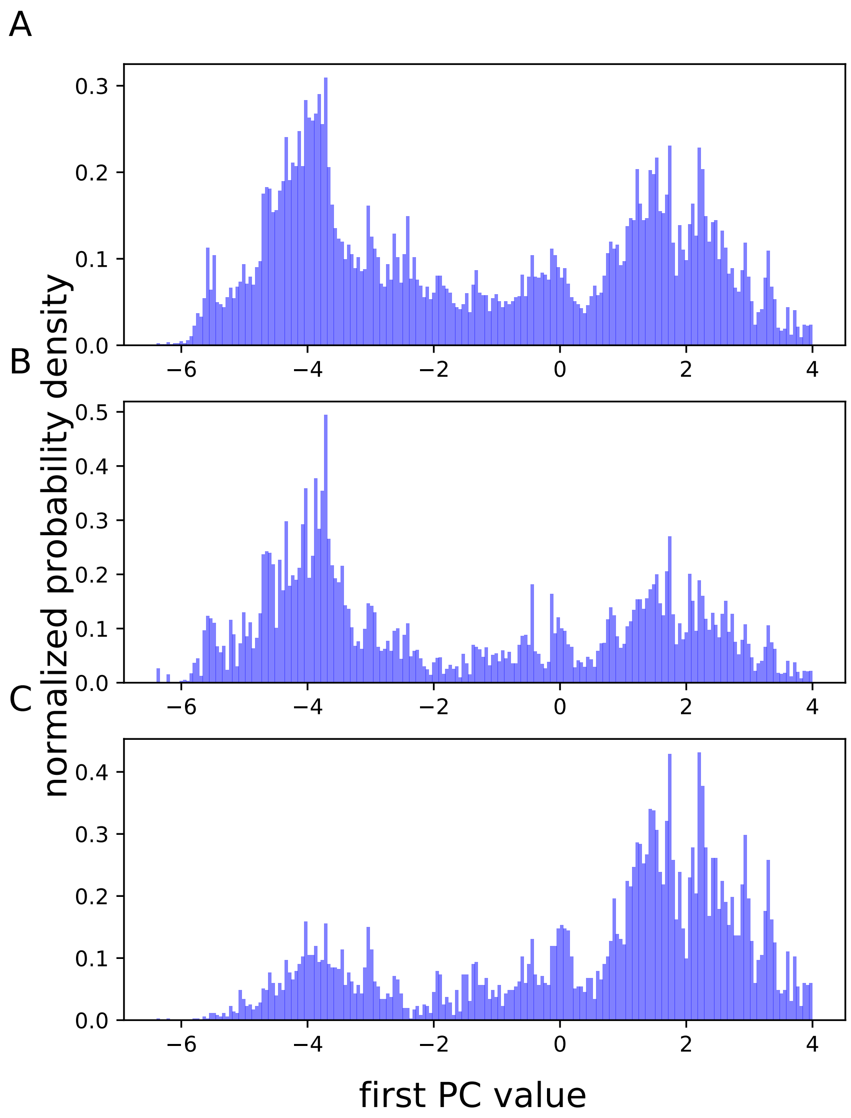


**Supplementary Figure 9.** Checking for undersampling bias in the data through interpolation of time. (A) PC1 linearly interpolated for each NGA for missing periods. (B) Each observation weighted based on its relative sparsity with respect to each NGA, so that observations in sparse periods weigh more. (C) Only data points from NGAs that span both Gaussians

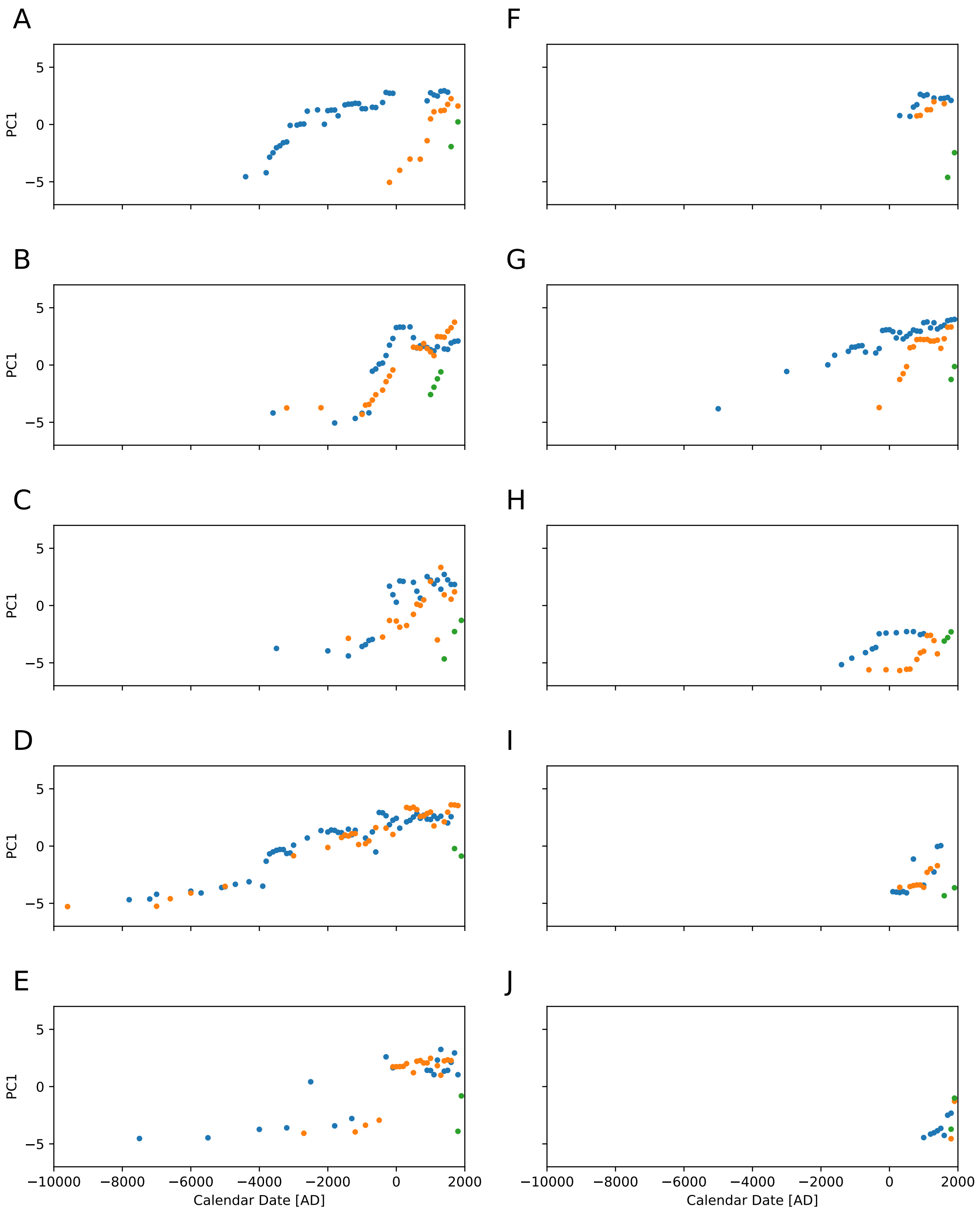


**Supplementary Figure 10.** Scores on PC1 as a function of time for 30 NGAs in 10 regions. Blue, orange, and green points are, respectively, NGAs in each region with early, intermediate, and late onsets of centralization. The regions are (left column): (A) Africa, (B) Europe, (C) Central Eurasia, (D) Southwest Asia, (E) South Asia; and (right column) (F) Southeast Asia, (G) East Asia, (H) North America, (I) South America, and (J) Oceania-Australia. The PC1 value is the mean across 20 imputations.

## Supplementary References


1. Turchin, P. *et al.* Seshat: The global history databank. *Cliodynamics* **6**, 77–107 (2015).
2. Turchin, P. *et al.* Quantitative historical analysis uncovers a single dimension of complexity that structures global variation in human social organization. *Proc. Natl. Acad. Sci.* **115**, E144–E151, DOI: 10.1073/pnas.1708800115 (2018). http://www.pnas.org/content/115/2/E144.full.pdf.
3. Whitehouse, H. *et al.* Complex societies precede moralizing gods throughout world history. *Nature* DOI: 10.1038/s41586-019-1043-4 (2019).
4. Turchin, P. Fitting Dynamic Regression Models to Seshat Data. *Cliodynamics* **9**, 25–58 (2018).
5. Turchin, P. *et al.* Evolutionary pathways to statehood: Old theories and new data. *SocArXiv Prepr. DOI: 10.31235/osf.io/h7tr6* (2018).
6. Peregrine, P. N. Toward a theory of recurrent social formations. In Sabloff, J. A. & Sabloff, P. L. W. (eds.) *The Emergence of Premodern States: New Perspectives on the Development of Complex Societies*, 271–295 (SFI Press, 2018).
