# Supplementary material for: Scale and information-processing thresholds in Holocene social evolution
Source: Nat Commun. 2020 May 14;11:2394. doi: 10.1038/s41467-020-16035-9 (PMC7224170; doi:10.1038/s41467-020-16035-9)
Supplement: Supplementary file 3 — Reporting Summary [file 41467_2020_16035_MOESM3_ESM.pdf]

nature research

Corresponding author(s): Timothy A. Kohler & David H. Wolpert

Last updated by author(s): Apr 3, 2020

Reporting Summary

Nature Research wishes to improve the reproducibility of the work that we publish. This form provides structure for consistency and transparency in reporting. For further information on Nature Research policies, see Authors & Referees and the Editorial Policy Checklist .

Statistics

For all statistical analyses, confirm that the following items are present in the figure legend, table legend, main text, or Methods section.

n/a	Confirmed	
☐	☒	The exact sample size (n) for each experimental group/condition, given as a discrete number and unit of measurement
☐	☒	A statement on whether measurements were taken from distinct samples or whether the same sample was measured repeatedly
☒	☐	The statistical test(s) used AND whether they are one- or two-sided<br>*Only common tests should be described solely by name; describe more complex techniques in the Methods section.*
☒	☐	A description of all covariates tested
☐	☒	A description of any assumptions or corrections, such as tests of normality and adjustment for multiple comparisons
☐	☒	A full description of the statistical parameters including central tendency (e.g. means) or other basic estimates (e.g. regression coefficient) AND variation (e.g. standard deviation) or associated estimates of uncertainty (e.g. confidence intervals)
☒	☐	For null hypothesis testing, the test statistic (e.g. F, t, r) with confidence intervals, effect sizes, degrees of freedom and P value noted<br>*Give P values as exact values whenever suitable.*
☐	☒	For Bayesian analysis, information on the choice of priors and Markov chain Monte Carlo settings
☒	☐	For hierarchical and complex designs, identification of the appropriate level for tests and full reporting of outcomes
☒	☐	Estimates of effect sizes (e.g. Cohen's d, Pearson's r), indicating how they were calculated

Our web collection on statistics for biologists contains articles on many of the points above.

Software and code

Policy information about availability of computer code

Data collection	No original data collection was performed for this study. We used data supplied by the Seshat project, as detailed in the paper.
Data analysis	Code for all figures and analyses is available through https://github.com/jaewshin/Holocene.

For manuscripts utilizing custom algorithms or software that are central to the research but not yet described in published literature, software must be made available to editors/reviewers. We strongly encourage code deposition in a community repository (e.g. GitHub). See the Nature Research guidelines for submitting code & software for further information.

Data

Policy information about availability of data

All manuscripts must include a data availability statement. This statement should provide the following information, where applicable:

- Accession codes, unique identifiers, or web links for publicly available datasets
- A list of figures that have associated raw data
- A description of any restrictions on data availability

This research employed data from the Seshat Databank (seshatdatabank.info) under Creative Commons Attribution Non-Commercial (CC By-NC SA) licensing which is available for download through http://seshatdatabank.info/datasets/.

Field-specific reporting

Please select the one below that is the best fit for your research. If you are not sure, read the appropriate sections before making your selection.

☐ Life sciences ☒ Behavioural & social sciences ☐ Ecological, evolutionary & environmental sciences

Behavioural & social sciences study design

All studies must disclose on these points even when the disclosure is negative.

Study description	Data are quantitative and worldwide in spatial scope, extending variably from the early Holocene to AD/CE 1900 depending on the region.
Research sample	We analyze the same 9 "Complexity Characteristics" (CCs) derived from 30 "Natural Geographic Areas" (NGAs) drawn from 10 world regions as analyzed by Turchin et al. (2017), since we strive to build on that study. The Seshat Global History Databank contains over 1500 variables from which Turchin et al. (2017) selected 51 which are relevant to describing growth in scale and in complexity of governing structure, political organization, and economic function. These were summarized by the 9 CCs.
Sampling strategy	The NGAs were sampled by Turchin et al. (2017) systematically from each of the 10 world regions, spanning six continents, so as to include one NGA in which complexity was developed early, one in which it developed late, and one which developed beginning at an intermediate date. K-fold cross-validation reported by Turchin et al. (2017:3-4) show overall ρ2 values calculated as an average of the ρ2 values weighted by the number of polities from which they are drawn ranging from 0.53 to 0.84, representing the success at predicting values for each CC when the world region from which that value was calculated was withheld from the sample.
Data collection	We use the same data reported by Turchin (2017), which were coded from the archaeological and historical literature for each of the 30 NGAs. Turchin et al. (2017:7-8) developed systematic procedures for dealing with missing data, uncertainty, and expert disagreement using multiple imputation, and here we also utilize the dataset containing their imputations (on which we provide some comment).
Timing	The earliest polity or quasipolity in any NGA dated to 9600 BC/BCE (on the Konya Plain). The most recent polities in the sample dated to AD/CE 1900 (in the Lowland Andes, Chuuk Islands, Yemeni Coastal Plain, Lena River Valley, Oro PNG, Southern China Hills, Kapuasi Basin, and Garo Hills NGAs). The average date across all the 414 datapoints in the sample is 120 BC/BCE.
Data exclusions	We did not exclude any data from the sample analyzed in Turchin (2017). They do not repoprt any data exclusions (but see below).
Non-participation	Obviously in a study such as this NGAs from which little archaeological or historical information is available have little or no chance of being in the sample.
Randomization	n/a

Reporting for specific materials, systems and methods

We require information from authors about some types of materials, experimental systems and methods used in many studies. Here, indicate whether each material, system or method listed is relevant to your study. If you are not sure if a list item applies to your research, read the appropriate section before selecting a response.

Materials & experimental systems

n/a	Involved in the study	
☒	☐	Antibodies
☒	☐	Eukaryotic cell lines
☒	☐	Palaeontology
☒	☐	Animals and other organisms
☒	☐	Human research participants
☒	☐	Clinical data

Methods

n/a	Involved in the study	
☒	☐	ChIP-seq
☒	☐	Flow cytometry
☒	☐	MRI-based neuroimaging
